# 2,2-Dimethyl-5-(2-naphthyl­amino­methyl­ene)-1,3-dioxane-4,6-dione

**DOI:** 10.1107/S1600536809038458

**Published:** 2009-09-30

**Authors:** Rui Li, Zhen-Yu Ding

**Affiliations:** a State Key Laboratory of Biotherapy, West China Hospital, Sichuan University, Chengdu 610041, People’s Republic of China

## Abstract

There are two unique mol­ecules in the asymmetric unit of the title compound, C_17_H_15_NO_4_, which are linked into chains *via* inter­molecular N—H⋯O and C—H⋯O inter­actions; the chains are linked *via* weak C—H⋯O inter­actions, forming a parallel sheet structure. The molecule is approximately planar, with dihedral angles of 19.91 (4) and 11.06 (4)° between the naphthyl ring and the amino­methyl­ene group, and between the amino­methyl­ene unit and the planar part of the dioxane ring, respectively. The dioxane ring adopts a half-boat conformation, with the C atom between the dioxane O atoms 0.595 (8) Å out of the plane through the remaining atoms. The mol­ecule has an intra­molecular N—H⋯O hydrogen bond which stabilizes the planar conformation.

## Related literature

For the synthesis of related compounds, see: Cassis *et al.* (1985 ▶). For the synthesis of related anti­tumor precursors, see: Ruchelman *et al.* (2003 ▶). For the crystal structures of other 5-aryl­amino­methyl­ene-2,2-dimethyl-1,3-dioxane-4,6-dione deriv­atives, see: Li *et al.* (2009*a* ▶,*b* ▶,*c* ▶).
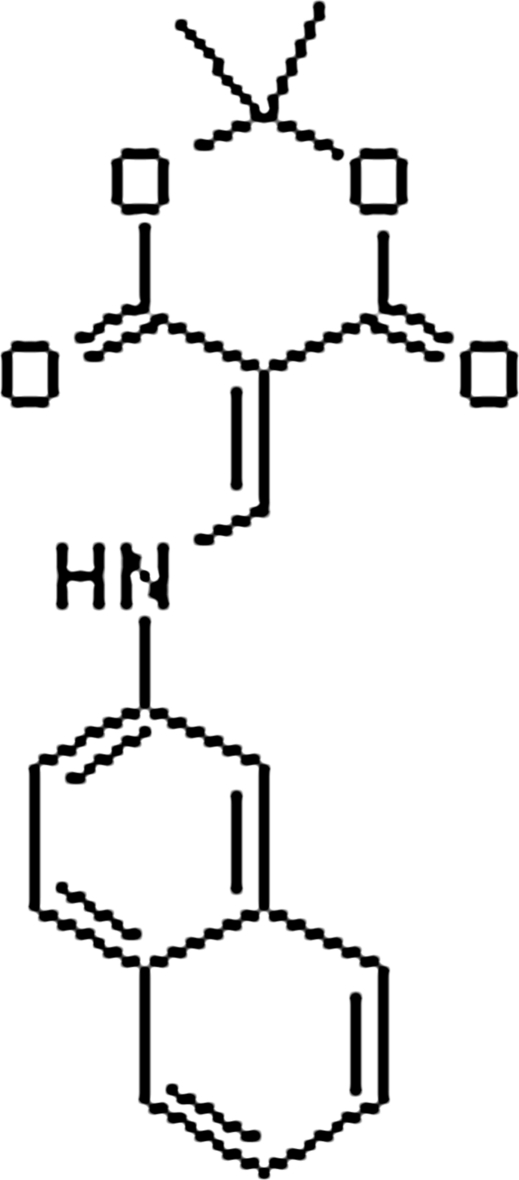


## Experimental

### 

#### Crystal data

C_17_H_15_NO_4_
*M* *_r_* = 297.30
Triclinic, 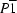

*a* = 10.6204 (17) Å
*b* = 11.8220 (19) Å
*c* = 12.0143 (19) Å
α = 78.237 (2)°
β = 86.786 (2)°
γ = 82.469 (2)°
*V* = 1463.4 (4) Å^3^
*Z* = 4
Mo *K*α radiation
μ = 0.10 mm^−1^
*T* = 153 K
0.25 × 0.22 × 0.20 mm

#### Data collection

Bruker SMART CCD area-detector diffractometer
Absorption correction: none
9205 measured reflections
6490 independent reflections
4313 reflections with *I* > 2σ(*I*)
*R*_int_ = 0.024

#### Refinement

*R*[*F* ^2^ > 2σ(*F* ^2^)] = 0.048
*wR*(*F* ^2^) = 0.127
*S* = 1.11
6490 reflections
410 parameters
H atoms treated by a mixture of independent and constrained refinement
Δρ_max_ = 0.23 e Å^−3^
Δρ_min_ = −0.19 e Å^−3^

### 

Data collection: *SMART* (Bruker, 2001 ▶); cell refinement: *SAINT* (Bruker, 2001 ▶); data reduction: *SAINT*; program(s) used to solve structure: *SHELXS97* (Sheldrick, 2008 ▶); program(s) used to refine structure: *SHELXL97* (Sheldrick, 2008 ▶); molecular graphics: *ORTEP-3* (Farrugia, 1997); software used to prepare material for publication: *SHELXL97* and *PLATON* (Spek, 2009 ▶).

## Supplementary Material

Crystal structure: contains datablocks I, global. DOI: 10.1107/S1600536809038458/bv2127sup1.cif

Structure factors: contains datablocks I. DOI: 10.1107/S1600536809038458/bv2127Isup2.hkl

Additional supplementary materials:  crystallographic information; 3D view; checkCIF report

## Figures and Tables

**Table 1:** Hydrogen-bond geometry (Å, °)

*D*—H⋯*A*	*D*—H	H⋯*A*	*D*⋯*A*	*D*—H⋯*A*
N1—H1⋯O3	0.92 (2)	2.11 (2)	2.7681 (18)	127.2 (16)
N1—H1⋯O7	0.92 (2)	2.35 (2)	3.184 (2)	150.9 (17)
N2—H2⋯O7	0.91 (2)	2.15 (2)	2.7858 (18)	126.7 (17)
N2—H2⋯O3	0.91 (2)	2.36 (2)	3.1893 (19)	152.9 (17)
C7—H7⋯O4	0.95	2.50	2.833 (2)	101
C14—H14⋯O4^i^	0.95	2.51	3.354 (2)	148
C24—H24⋯O8	0.95	2.45	2.804 (2)	102
C26—H26⋯O3	0.95	2.46	3.2569 (19)	142

Symmetry code: (i) 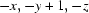.

**Fig. 1.**
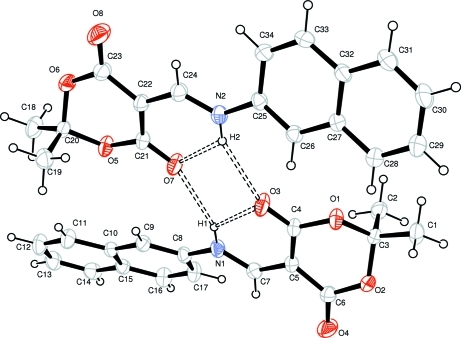
The molecular structure of the title compound, with displacement ellipsoids drawn at the 30% probability level.

## supplementary crystallographic information

### Comment

5-Arylaminomethylene-2,2-dimethyl-1,3-dioxane-4,6-diones are key intermediates and can be used to synthesize 4(1*H*)quinolone derivatives by thermolysis (Cassis *et al.*, 1985) which can be used as precursors for anticancer agents, anti-malarial agents and reversible (H+/K+) ATPase inhibitors (Ruchelman *et al.*,2003). The molecule (Fig. 1) is approximately planar with dihedral angles of 19.91 (4)° and 11.06 (4)° between the benzyl ring and the aminomethylene group, and between the aminomethylene unit and the planar part of the dioxane ring, respectively. In addition, the dioxane ring of the title compound adopts a half-boat conformation, in which the C atom between the dioxane oxygen atoms is -0.595 (8) Å out-of-plane. The intramolecular N—H···O hydrogen bond (Table 1) is stabilizing the planar conformation in the molecule.

### Experimental

An ethanol solution of 2,2-dimethyl-1,3-dioxane-4,6-dione (1.44 g, 0.01 mol) with methylorthoformate (1.27 g, 0.012 mol) was heated to reflux for 2 h, then the arylamine (1.32 g, 0.01 mol) was added into the solution. The mixture was heated under reflux for another 10 h and then filtered. Single crystals were obtained from the filtrate after 2 days.

### Refinement

The imino H atom was located in a difference Fourier map and refined isotropically. Other H atoms were positioned geometrically with C—H = 0.93 (aromatic) or 0.96 Å (methyl), and refined using a riding model with *U*_ĩso_(H) = 1.5*U*_eq_(C) for methyl and 1.2*U*_eq_(C) for the others.

### Crystal data

**Table:**

C_17_H_15_NO_4_	*Z* = 4
*M**_r_* = 297.30	*F*(000) = 624
Triclinic, *P*1	*D*_x_ = 1.349 Mg m^−^^3^
Hall symbol: -P 1	Mo *K*α radiation, λ = 0.71073 Å
*a* = 10.6204 (17) Å	Cell parameters from 2937 reflections
*b* = 11.8220 (19) Å	θ = 2.5–27.5°
*c* = 12.0143 (19) Å	µ = 0.10 mm^−^^1^
α = 78.237 (2)°	*T* = 153 K
β = 86.786 (2)°	Block, colourless
γ = 82.469 (2)°	0.25 × 0.22 × 0.20 mm
*V* = 1463.4 (4) Å^3^	

### Data collection

**Table:**

Bruker SMART CCD area-detector diffractometer	4313 reflections with *I* > 2σ(*I*)
Radiation source: fine-focus sealed tube	*R*_int_ = 0.024
graphite	θ_max_ = 27.6°, θ_min_ = 2.5°
φ and ω scans	*h* = −10→13
9205 measured reflections	*k* = −15→14
6490 independent reflections	*l* = −15→14

### Refinement

**Table:**

Refinement on *F*^2^	Secondary atom site location: difference Fourier map
Least-squares matrix: full	Hydrogen site location: mixed
*R*[*F*^2^ > 2σ(*F*^2^)] = 0.048	H atoms treated by a mixture of independent and constrained refinement
*wR*(*F*^2^) = 0.127	*w* = 1/[σ^2^(*F*_o_^2^) + (0.0403*P*)^2^ + 0.2303*P*] where *P* = (*F*_o_^2^ + 2*F*_c_^2^)/3
*S* = 1.11	(Δ/σ)_max_ < 0.001
6490 reflections	Δρ_max_ = 0.23 e Å^−^^3^
410 parameters	Δρ_min_ = −0.19 e Å^−^^3^
0 restraints	Extinction correction: *SHELXL97* (Sheldrick, 2008), Fc^*^=kFc[1+0.001xFc^2^λ^3^/sin(2θ)]^-1/4^
Primary atom site location: structure-invariant direct methods	Extinction coefficient: 0.045 (2)

### Special details

**Table:**

Geometry. All e.s.d.'s (except the e.s.d. in the dihedral angle between two l.s. planes) are estimated using the full covariance matrix. The cell e.s.d.'s are taken into account individually in the estimation of e.s.d.'s in distances, angles and torsion angles; correlations between e.s.d.'s in cell parameters are only used when they are defined by crystal symmetry. An approximate (isotropic) treatment of cell e.s.d.'s is used for estimating e.s.d.'s involving l.s. planes.
Refinement. Refinement of *F*^2^ against ALL reflections. The weighted *R*-factor *wR* and goodness of fit *S* are based on *F*^2^, conventional *R*-factors *R* are based on *F*, with *F* set to zero for negative *F*^2^. The threshold expression of *F*^2^ > σ(*F*^2^) is used only for calculating *R*-factors(gt) *etc*. and is not relevant to the choice of reflections for refinement. *R*-factors based on *F*^2^ are statistically about twice as large as those based on *F*, and *R*- factors based on ALL data will be even larger.

### Fractional atomic coordinates and isotropic or equivalent isotropic displacement parameters (Å^2^)

**Table:**

	*x*	*y*	*z*	*U*_iso_*/*U*_eq_	
O1	0.09234 (11)	0.24925 (9)	0.63633 (8)	0.0467 (3)	
O2	−0.06511 (10)	0.17438 (10)	0.55278 (9)	0.0477 (3)	
O3	0.17092 (13)	0.40987 (10)	0.55022 (9)	0.0590 (4)	
O4	−0.13735 (13)	0.25516 (13)	0.38302 (11)	0.0739 (4)	
N1	0.13306 (14)	0.48532 (12)	0.31953 (12)	0.0455 (3)	
H1	0.1759 (19)	0.4990 (18)	0.3788 (17)	0.078 (7)*	
C1	0.01090 (19)	0.07785 (16)	0.73214 (15)	0.0578 (5)	
H1A	−0.0588	0.1241	0.7658	0.087*	
H1B	0.0839	0.0615	0.7819	0.087*	
H1C	−0.0170	0.0044	0.7235	0.087*	
C2	0.15032 (16)	0.07683 (15)	0.55656 (15)	0.0534 (4)	
H2A	0.1202	0.0048	0.5463	0.080*	
H2B	0.2271	0.0577	0.6016	0.080*	
H2C	0.1698	0.1239	0.4820	0.080*	
C3	0.04856 (15)	0.14458 (13)	0.61769 (13)	0.0406 (4)	
C4	0.10548 (16)	0.33504 (13)	0.54327 (13)	0.0413 (4)	
C5	0.03603 (15)	0.33192 (13)	0.44535 (13)	0.0413 (4)	
C6	−0.06033 (16)	0.25392 (15)	0.45320 (14)	0.0474 (4)	
C7	0.05448 (15)	0.40537 (14)	0.34294 (13)	0.0450 (4)	
H7	0.0050	0.3977	0.2820	0.054*	
C8	0.15665 (15)	0.54980 (13)	0.20834 (12)	0.0412 (4)	
C9	0.19882 (15)	0.65634 (14)	0.19600 (13)	0.0425 (4)	
H9	0.2116	0.6864	0.2615	0.051*	
C10	0.22353 (14)	0.72218 (13)	0.08622 (13)	0.0407 (4)	
C11	0.26546 (18)	0.83343 (16)	0.06954 (15)	0.0562 (5)	
H11	0.2766	0.8665	0.1335	0.067*	
C12	0.2901 (2)	0.89383 (17)	−0.03759 (17)	0.0669 (6)	
H12	0.3186	0.9682	−0.0474	0.080*	
C13	0.27356 (19)	0.84666 (17)	−0.13264 (16)	0.0635 (5)	
H13	0.2916	0.8891	−0.2066	0.076*	
C14	0.23192 (17)	0.74096 (17)	−0.12038 (14)	0.0543 (5)	
H14	0.2207	0.7102	−0.1858	0.065*	
C15	0.20497 (15)	0.67593 (14)	−0.01052 (13)	0.0428 (4)	
C16	0.16170 (17)	0.56607 (15)	0.00616 (14)	0.0525 (4)	
H16	0.1483	0.5345	−0.0582	0.063*	
C17	0.13853 (17)	0.50381 (15)	0.11220 (14)	0.0518 (4)	
H17	0.1102	0.4294	0.1212	0.062*	
O5	0.42844 (11)	0.73498 (10)	0.35805 (9)	0.0496 (3)	
O6	0.57288 (11)	0.81921 (10)	0.44864 (10)	0.0524 (3)	
O7	0.33468 (12)	0.58478 (10)	0.44833 (10)	0.0573 (3)	
O8	0.62505 (15)	0.75181 (14)	0.62628 (12)	0.0866 (5)	
N2	0.37240 (13)	0.50869 (12)	0.68054 (11)	0.0445 (3)	
H2	0.3243 (19)	0.4993 (18)	0.6238 (17)	0.078 (7)*	
C18	0.5313 (2)	0.89190 (17)	0.25626 (15)	0.0648 (5)	
H18A	0.5608	0.9662	0.2598	0.097*	
H18B	0.4678	0.9046	0.1974	0.097*	
H18C	0.6035	0.8375	0.2377	0.097*	
C19	0.36396 (17)	0.92034 (15)	0.40890 (17)	0.0641 (5)	
H19A	0.3331	0.8835	0.4843	0.096*	
H19B	0.2951	0.9340	0.3550	0.096*	
H19C	0.3927	0.9947	0.4131	0.096*	
C20	0.47273 (15)	0.84181 (14)	0.36956 (13)	0.0427 (4)	
C21	0.40282 (15)	0.65788 (13)	0.45482 (13)	0.0409 (4)	
C22	0.46411 (15)	0.66640 (14)	0.55547 (13)	0.0423 (4)	
C23	0.55744 (17)	0.74667 (16)	0.55030 (15)	0.0527 (4)	
C24	0.44707 (16)	0.59171 (14)	0.65771 (14)	0.0469 (4)	
H24	0.4948	0.6011	0.7191	0.056*	
C25	0.36078 (15)	0.43352 (13)	0.78831 (12)	0.0401 (4)	
C26	0.25699 (15)	0.37528 (13)	0.81217 (12)	0.0401 (4)	
H26	0.1945	0.3850	0.7563	0.048*	
C27	0.24066 (15)	0.30061 (13)	0.91865 (12)	0.0393 (4)	
C28	0.13167 (17)	0.24240 (15)	0.94677 (15)	0.0519 (4)	
H28	0.0682	0.2506	0.8920	0.062*	
C29	0.11669 (19)	0.17443 (17)	1.05196 (16)	0.0656 (5)	
H29	0.0429	0.1360	1.0698	0.079*	
C30	0.2093 (2)	0.16097 (18)	1.13367 (16)	0.0681 (6)	
H30	0.1974	0.1139	1.2067	0.082*	
C31	0.31577 (19)	0.21456 (16)	1.10953 (15)	0.0599 (5)	
H31	0.3782	0.2040	1.1656	0.072*	
C32	0.33477 (15)	0.28624 (14)	1.00126 (13)	0.0443 (4)	
C33	0.44196 (16)	0.34644 (16)	0.97260 (15)	0.0554 (5)	
H33	0.5062	0.3371	1.0268	0.066*	
C34	0.45594 (16)	0.41743 (16)	0.86962 (15)	0.0538 (5)	
H34	0.5296	0.4562	0.8523	0.065*	

### Atomic displacement parameters (Å^2^)

**Table:**

	*U*^11^	*U*^22^	*U*^33^	*U*^12^	*U*^13^	*U*^23^
O1	0.0684 (8)	0.0379 (6)	0.0360 (6)	−0.0205 (5)	−0.0011 (5)	−0.0034 (5)
O2	0.0396 (6)	0.0470 (7)	0.0538 (7)	−0.0157 (5)	−0.0014 (5)	0.0026 (5)
O3	0.0873 (9)	0.0488 (7)	0.0466 (7)	−0.0372 (7)	−0.0040 (6)	−0.0040 (6)
O4	0.0644 (9)	0.0939 (11)	0.0631 (8)	−0.0350 (8)	−0.0208 (7)	0.0064 (8)
N1	0.0535 (9)	0.0405 (8)	0.0391 (8)	−0.0105 (6)	0.0003 (6)	0.0024 (6)
C1	0.0670 (12)	0.0452 (10)	0.0570 (11)	−0.0183 (9)	0.0054 (9)	0.0046 (8)
C2	0.0477 (10)	0.0421 (10)	0.0709 (12)	−0.0088 (8)	0.0029 (9)	−0.0115 (9)
C3	0.0421 (9)	0.0341 (8)	0.0457 (9)	−0.0134 (7)	−0.0018 (7)	−0.0024 (7)
C4	0.0536 (10)	0.0342 (8)	0.0362 (8)	−0.0135 (7)	0.0037 (7)	−0.0036 (6)
C5	0.0444 (9)	0.0373 (8)	0.0403 (8)	−0.0087 (7)	−0.0002 (7)	−0.0011 (7)
C6	0.0444 (9)	0.0512 (10)	0.0446 (9)	−0.0110 (8)	−0.0030 (7)	−0.0012 (8)
C7	0.0450 (9)	0.0456 (9)	0.0417 (9)	−0.0063 (7)	−0.0036 (7)	−0.0012 (7)
C8	0.0452 (9)	0.0379 (9)	0.0361 (8)	−0.0043 (7)	0.0025 (7)	0.0010 (7)
C9	0.0476 (9)	0.0441 (9)	0.0353 (8)	−0.0095 (7)	−0.0031 (7)	−0.0037 (7)
C10	0.0401 (9)	0.0402 (9)	0.0388 (8)	−0.0062 (7)	−0.0012 (6)	−0.0002 (7)
C11	0.0656 (12)	0.0491 (10)	0.0535 (10)	−0.0188 (9)	0.0028 (9)	−0.0034 (8)
C12	0.0770 (14)	0.0516 (11)	0.0663 (13)	−0.0209 (10)	0.0066 (11)	0.0077 (10)
C13	0.0665 (13)	0.0625 (12)	0.0494 (11)	−0.0103 (10)	0.0036 (9)	0.0175 (9)
C14	0.0540 (11)	0.0664 (12)	0.0374 (9)	−0.0041 (9)	0.0004 (8)	−0.0011 (8)
C15	0.0413 (9)	0.0462 (9)	0.0376 (8)	−0.0031 (7)	0.0001 (7)	−0.0022 (7)
C16	0.0683 (12)	0.0515 (10)	0.0402 (9)	−0.0112 (9)	0.0065 (8)	−0.0147 (8)
C17	0.0689 (12)	0.0381 (9)	0.0501 (10)	−0.0131 (8)	0.0101 (8)	−0.0118 (8)
O5	0.0693 (8)	0.0437 (7)	0.0390 (6)	−0.0249 (6)	0.0039 (5)	−0.0060 (5)
O6	0.0456 (7)	0.0548 (7)	0.0549 (7)	−0.0212 (6)	−0.0028 (5)	0.0036 (6)
O7	0.0753 (9)	0.0505 (7)	0.0513 (7)	−0.0328 (7)	−0.0010 (6)	−0.0066 (6)
O8	0.0945 (11)	0.1021 (12)	0.0665 (9)	−0.0550 (10)	−0.0292 (8)	0.0102 (8)
N2	0.0478 (8)	0.0442 (8)	0.0391 (8)	−0.0097 (6)	0.0028 (6)	−0.0011 (6)
C18	0.0769 (13)	0.0625 (12)	0.0536 (11)	−0.0314 (11)	0.0089 (10)	0.0032 (9)
C19	0.0556 (11)	0.0437 (10)	0.0898 (15)	−0.0043 (9)	0.0040 (10)	−0.0085 (10)
C20	0.0454 (9)	0.0363 (8)	0.0474 (9)	−0.0150 (7)	−0.0019 (7)	−0.0039 (7)
C21	0.0474 (9)	0.0360 (8)	0.0398 (8)	−0.0116 (7)	0.0072 (7)	−0.0068 (7)
C22	0.0436 (9)	0.0407 (9)	0.0415 (9)	−0.0106 (7)	0.0021 (7)	−0.0036 (7)
C23	0.0522 (10)	0.0569 (11)	0.0488 (10)	−0.0182 (9)	−0.0052 (8)	−0.0019 (8)
C24	0.0478 (10)	0.0474 (10)	0.0448 (9)	−0.0101 (8)	0.0012 (7)	−0.0052 (8)
C25	0.0424 (9)	0.0389 (8)	0.0361 (8)	−0.0035 (7)	0.0032 (7)	−0.0030 (7)
C26	0.0450 (9)	0.0404 (9)	0.0345 (8)	−0.0057 (7)	−0.0034 (6)	−0.0057 (7)
C27	0.0422 (9)	0.0370 (8)	0.0376 (8)	−0.0045 (7)	−0.0004 (7)	−0.0052 (7)
C28	0.0501 (10)	0.0479 (10)	0.0551 (10)	−0.0120 (8)	−0.0050 (8)	0.0003 (8)
C29	0.0589 (12)	0.0595 (12)	0.0696 (13)	−0.0179 (10)	0.0024 (10)	0.0133 (10)
C30	0.0727 (13)	0.0635 (13)	0.0547 (11)	−0.0132 (10)	0.0011 (10)	0.0217 (9)
C31	0.0640 (12)	0.0604 (12)	0.0470 (10)	−0.0051 (10)	−0.0118 (9)	0.0102 (9)
C32	0.0460 (9)	0.0405 (9)	0.0419 (9)	−0.0017 (7)	−0.0043 (7)	0.0007 (7)
C33	0.0463 (10)	0.0602 (11)	0.0542 (10)	−0.0065 (8)	−0.0162 (8)	0.0056 (9)
C34	0.0390 (9)	0.0572 (11)	0.0594 (11)	−0.0111 (8)	−0.0048 (8)	0.0063 (9)

### Geometric parameters (Å, °)

**Table:**

O1—C4	1.3621 (17)	O5—C21	1.3616 (17)
O1—C3	1.4399 (17)	O5—C20	1.4386 (18)
O2—C6	1.3657 (18)	O6—C23	1.3570 (19)
O2—C3	1.4393 (18)	O6—C20	1.4286 (19)
O3—C4	1.2126 (18)	O7—C21	1.2143 (18)
O4—C6	1.2039 (19)	O8—C23	1.208 (2)
N1—C7	1.319 (2)	N2—C24	1.318 (2)
N1—C8	1.4232 (18)	N2—C25	1.4223 (19)
N1—H1	0.92 (2)	N2—H2	0.91 (2)
C1—C3	1.501 (2)	C18—C20	1.504 (2)
C1—H1A	0.9800	C18—H18A	0.9800
C1—H1B	0.9800	C18—H18B	0.9800
C1—H1C	0.9800	C18—H18C	0.9800
C2—C3	1.508 (2)	C19—C20	1.502 (2)
C2—H2A	0.9800	C19—H19A	0.9800
C2—H2B	0.9800	C19—H19B	0.9800
C2—H2C	0.9800	C19—H19C	0.9800
C4—C5	1.432 (2)	C21—C22	1.432 (2)
C5—C7	1.374 (2)	C22—C24	1.377 (2)
C5—C6	1.452 (2)	C22—C23	1.450 (2)
C7—H7	0.9500	C24—H24	0.9500
C8—C9	1.369 (2)	C25—C26	1.362 (2)
C8—C17	1.405 (2)	C25—C34	1.413 (2)
C9—C10	1.418 (2)	C26—C27	1.414 (2)
C9—H9	0.9500	C26—H26	0.9500
C10—C15	1.415 (2)	C27—C28	1.413 (2)
C10—C11	1.416 (2)	C27—C32	1.418 (2)
C11—C12	1.368 (2)	C28—C29	1.366 (2)
C11—H11	0.9500	C28—H28	0.9500
C12—C13	1.398 (3)	C29—C30	1.400 (3)
C12—H12	0.9500	C29—H29	0.9500
C13—C14	1.357 (3)	C30—C31	1.357 (3)
C13—H13	0.9500	C30—H30	0.9500
C14—C15	1.420 (2)	C31—C32	1.420 (2)
C14—H14	0.9500	C31—H31	0.9500
C15—C16	1.406 (2)	C32—C33	1.410 (2)
C16—C17	1.362 (2)	C33—C34	1.359 (2)
C16—H16	0.9500	C33—H33	0.9500
C17—H17	0.9500	C34—H34	0.9500
			
C4—O1—C3	117.01 (11)	C21—O5—C20	117.93 (12)
C6—O2—C3	117.24 (12)	C23—O6—C20	118.76 (12)
C7—N1—C8	124.07 (15)	C24—N2—C25	124.62 (15)
C7—N1—H1	117.5 (13)	C24—N2—H2	117.8 (13)
C8—N1—H1	118.4 (13)	C25—N2—H2	117.6 (13)
C3—C1—H1A	109.5	C20—C18—H18A	109.5
C3—C1—H1B	109.5	C20—C18—H18B	109.5
H1A—C1—H1B	109.5	H18A—C18—H18B	109.5
C3—C1—H1C	109.5	C20—C18—H18C	109.5
H1A—C1—H1C	109.5	H18A—C18—H18C	109.5
H1B—C1—H1C	109.5	H18B—C18—H18C	109.5
C3—C2—H2A	109.5	C20—C19—H19A	109.5
C3—C2—H2B	109.5	C20—C19—H19B	109.5
H2A—C2—H2B	109.5	H19A—C19—H19B	109.5
C3—C2—H2C	109.5	C20—C19—H19C	109.5
H2A—C2—H2C	109.5	H19A—C19—H19C	109.5
H2B—C2—H2C	109.5	H19B—C19—H19C	109.5
O1—C3—O2	109.46 (12)	O6—C20—O5	110.64 (12)
O1—C3—C1	106.82 (13)	O6—C20—C19	110.09 (14)
O2—C3—C1	106.47 (13)	O5—C20—C19	109.23 (13)
O1—C3—C2	110.33 (12)	O6—C20—C18	105.89 (13)
O2—C3—C2	110.52 (13)	O5—C20—C18	106.83 (13)
C1—C3—C2	113.07 (14)	C19—C20—C18	114.08 (15)
O3—C4—O1	118.26 (14)	O7—C21—O5	117.95 (14)
O3—C4—C5	124.93 (14)	O7—C21—C22	125.19 (14)
O1—C4—C5	116.77 (13)	O5—C21—C22	116.78 (13)
C7—C5—C4	121.39 (14)	C24—C22—C21	121.87 (15)
C7—C5—C6	118.04 (15)	C24—C22—C23	117.21 (15)
C4—C5—C6	120.52 (14)	C21—C22—C23	120.61 (14)
O4—C6—O2	117.83 (15)	O8—C23—O6	117.59 (16)
O4—C6—C5	126.67 (15)	O8—C23—C22	126.17 (16)
O2—C6—C5	115.46 (14)	O6—C23—C22	116.19 (15)
N1—C7—C5	127.43 (16)	N2—C24—C22	127.68 (16)
N1—C7—H7	116.3	N2—C24—H24	116.2
C5—C7—H7	116.3	C22—C24—H24	116.2
C9—C8—C17	120.32 (14)	C26—C25—C34	119.89 (14)
C9—C8—N1	119.35 (14)	C26—C25—N2	119.13 (14)
C17—C8—N1	120.33 (14)	C34—C25—N2	120.96 (14)
C8—C9—C10	120.49 (14)	C25—C26—C27	121.14 (14)
C8—C9—H9	119.8	C25—C26—H26	119.4
C10—C9—H9	119.8	C27—C26—H26	119.4
C15—C10—C11	118.51 (14)	C28—C27—C26	122.14 (14)
C15—C10—C9	119.14 (14)	C28—C27—C32	118.80 (14)
C11—C10—C9	122.35 (15)	C26—C27—C32	119.04 (14)
C12—C11—C10	120.78 (17)	C29—C28—C27	120.60 (17)
C12—C11—H11	119.6	C29—C28—H28	119.7
C10—C11—H11	119.6	C27—C28—H28	119.7
C11—C12—C13	120.34 (18)	C28—C29—C30	120.53 (17)
C11—C12—H12	119.8	C28—C29—H29	119.7
C13—C12—H12	119.8	C30—C29—H29	119.7
C14—C13—C12	120.71 (16)	C31—C30—C29	120.61 (17)
C14—C13—H13	119.6	C31—C30—H30	119.7
C12—C13—H13	119.6	C29—C30—H30	119.7
C13—C14—C15	120.52 (17)	C30—C31—C32	120.60 (17)
C13—C14—H14	119.7	C30—C31—H31	119.7
C15—C14—H14	119.7	C32—C31—H31	119.7
C16—C15—C10	118.43 (14)	C33—C32—C27	118.13 (14)
C16—C15—C14	122.44 (15)	C33—C32—C31	122.99 (16)
C10—C15—C14	119.13 (15)	C27—C32—C31	118.86 (15)
C17—C16—C15	121.73 (15)	C34—C33—C32	121.85 (16)
C17—C16—H16	119.1	C34—C33—H33	119.1
C15—C16—H16	119.1	C32—C33—H33	119.1
C16—C17—C8	119.88 (15)	C33—C34—C25	119.93 (15)
C16—C17—H17	120.1	C33—C34—H34	120.0
C8—C17—H17	120.1	C25—C34—H34	120.0
			
C4—O1—C3—O2	−51.22 (17)	C23—O6—C20—O5	−47.53 (19)
C4—O1—C3—C1	−166.12 (14)	C23—O6—C20—C19	73.30 (18)
C4—O1—C3—C2	70.61 (17)	C23—O6—C20—C18	−162.94 (15)
C6—O2—C3—O1	52.86 (17)	C21—O5—C20—O6	47.95 (18)
C6—O2—C3—C1	167.99 (14)	C21—O5—C20—C19	−73.39 (18)
C6—O2—C3—C2	−68.85 (17)	C21—O5—C20—C18	162.76 (14)
C3—O1—C4—O3	−161.61 (15)	C20—O5—C21—O7	160.40 (14)
C3—O1—C4—C5	20.7 (2)	C20—O5—C21—C22	−22.6 (2)
O3—C4—C5—C7	10.6 (3)	O7—C21—C22—C24	−2.1 (3)
O1—C4—C5—C7	−171.89 (14)	O5—C21—C22—C24	−178.83 (14)
O3—C4—C5—C6	−166.65 (17)	O7—C21—C22—C23	171.31 (17)
O1—C4—C5—C6	10.8 (2)	O5—C21—C22—C23	−5.5 (2)
C3—O2—C6—O4	158.74 (16)	C20—O6—C23—O8	−161.02 (17)
C3—O2—C6—C5	−23.5 (2)	C20—O6—C23—C22	21.5 (2)
C7—C5—C6—O4	−9.3 (3)	C24—C22—C23—O8	2.6 (3)
C4—C5—C6—O4	168.11 (18)	C21—C22—C23—O8	−171.02 (19)
C7—C5—C6—O2	173.17 (14)	C24—C22—C23—O6	179.83 (15)
C4—C5—C6—O2	−9.4 (2)	C21—C22—C23—O6	6.2 (2)
C8—N1—C7—C5	173.10 (16)	C25—N2—C24—C22	179.14 (16)
C4—C5—C7—N1	−0.2 (3)	C21—C22—C24—N2	−3.1 (3)
C6—C5—C7—N1	177.14 (16)	C23—C22—C24—N2	−176.69 (16)
C7—N1—C8—C9	155.71 (16)	C24—N2—C25—C26	161.00 (16)
C7—N1—C8—C17	−25.2 (2)	C24—N2—C25—C34	−19.9 (2)
C17—C8—C9—C10	0.6 (2)	C34—C25—C26—C27	1.6 (2)
N1—C8—C9—C10	179.75 (14)	N2—C25—C26—C27	−179.29 (14)
C8—C9—C10—C15	−0.5 (2)	C25—C26—C27—C28	177.77 (16)
C8—C9—C10—C11	179.06 (16)	C25—C26—C27—C32	−0.3 (2)
C15—C10—C11—C12	−1.4 (3)	C26—C27—C28—C29	−177.48 (17)
C9—C10—C11—C12	179.07 (17)	C32—C27—C28—C29	0.6 (3)
C10—C11—C12—C13	0.3 (3)	C27—C28—C29—C30	−0.2 (3)
C11—C12—C13—C14	0.5 (3)	C28—C29—C30—C31	−0.4 (3)
C12—C13—C14—C15	−0.3 (3)	C29—C30—C31—C32	0.6 (3)
C11—C10—C15—C16	−179.17 (16)	C28—C27—C32—C33	−178.95 (16)
C9—C10—C15—C16	0.4 (2)	C26—C27—C32—C33	−0.8 (2)
C11—C10—C15—C14	1.6 (2)	C28—C27—C32—C31	−0.4 (2)
C9—C10—C15—C14	−178.87 (15)	C26—C27—C32—C31	177.72 (15)
C13—C14—C15—C16	−179.98 (17)	C30—C31—C32—C33	178.27 (19)
C13—C14—C15—C10	−0.7 (3)	C30—C31—C32—C27	−0.2 (3)
C10—C15—C16—C17	−0.5 (3)	C27—C32—C33—C34	0.6 (3)
C14—C15—C16—C17	178.76 (17)	C31—C32—C33—C34	−177.87 (18)
C15—C16—C17—C8	0.6 (3)	C32—C33—C34—C25	0.7 (3)
C9—C8—C17—C16	−0.7 (3)	C26—C25—C34—C33	−1.9 (3)
N1—C8—C17—C16	−179.82 (16)	N2—C25—C34—C33	179.09 (16)

### Hydrogen-bond geometry (Å, °)

**Table:**

*D*—H···*A*	*D*—H	H···*A*	*D*···*A*	*D*—H···*A*
N1—H1···O3	0.92 (2)	2.11 (2)	2.7681 (18)	127.2 (16)
N1—H1···O7	0.92 (2)	2.35 (2)	3.184 (2)	150.9 (17)
N2—H2···O7	0.91 (2)	2.15 (2)	2.7858 (18)	126.7 (17)
N2—H2···O3	0.91 (2)	2.36 (2)	3.1893 (19)	152.9 (17)
C7—H7···O4	0.95	2.50	2.833 (2)	101
C14—H14···O4^i^	0.95	2.51	3.354 (2)	148
C24—H24···O8	0.95	2.45	2.804 (2)	102
C26—H26···O3	0.95	2.46	3.2569 (19)	142

Symmetry codes: (i) −*x*, −*y*+1, −*z*.
